# T‐cell response to phytohemagglutinin in the interferon‐γ release assay as a potential biomarker for the response to immune checkpoint inhibitors in patients with non‐small cell lung cancer

**DOI:** 10.1111/1759-7714.13978

**Published:** 2021-05-04

**Authors:** Chisato Kamimaki, Nobuaki Kobayashi, Momo Hirata, Kohei Somekawa, Nobuhiko Fukuda, Sousuke Kubo, Seigo Katakura, Shuhei Teranishi, Keisuke Watanabe, Nobuyuki Horita, Yu Hara, Masaki Yamamoto, Makoto Kudo, Hongmei Piao, Takeshi Kaneko

**Affiliations:** ^1^ Department of Pulmonology Yokohama City University Graduate School of Medicine Yokohama Japan; ^2^ Department of Respiratory Medicine Yokohama City Medical Center Yokohama Japan; ^3^ Department of Respiratory Medicine Yanbian University Hospital Yanji China

**Keywords:** immune checkpoint inhibitor, interferon‐γ, interferon‐γ‐releasing assay, lung cancer, T‐SPOT.TB

## Abstract

**Background:**

Immune checkpoint inhibitors are a standard treatment for advanced lung cancer, although it remains important to identify biomarkers that can accurately predict treatment response. Immune checkpoint inhibitors enhance the antitumor T‐cell response, and interferon‐γ plays an important role in this process. Therefore, this study evaluated whether the number of interferon‐γ‐releasing peripheral T cells after phytohemagglutinin stimulation in the interferon‐γ release assay might act as a biomarker for the response of non‐small cell lung cancer to immune checkpoint inhibitor treatment.

**Methods:**

Data were retrospectively collected regarding 74 patients with non‐small cell lung cancer who had received immune checkpoint inhibitors. Pretreatment screening tests had been performed using the T‐SPOT.TB assay, which quantifies the number of interferon‐γ‐releasing T cells (as immunospots) in response to phytohemagglutinin and tuberculosis‐specific antigen stimulation. Clinical factors and the number of spots in the T‐SPOT fields were evaluated for associations with patient outcomes. The median number of spots was used to categorize patients as having high or low values, and the two groups were compared.

**Results:**

Relative to patients with a low ratio, patients with a high ratio of phytohemagglutinin/tuberculosis‐specific antigen spots (i.e. more responsive T cells) had significantly better progression‐free survival after immune checkpoint inhibitor treatment. When we only considered patients with negative T‐SPOT results, a high number of phytohemagglutinin‐stimulated spots corresponded to significantly longer progression‐free survival.

**Conclusion:**

The T‐SPOT.TB assay can be used to quantify the number of immunospots in response to antigen stimulation, which may predict the response to immune checkpoint inhibitors in patients with non‐small cell lung cancer.

## Graphical abstract

This study evaluated whether the T‐SPOT test could be used to predict the response to immune checkpoint inhibitor treatment in patients with non‐small cell lung cancer. Among patients with negative T‐SPOT results, a high number of phytohemagglutinin‐stimulated spots was associated with longer progression‐free survival.

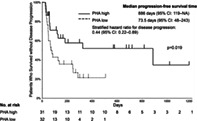

## BACKGROUND

Lung cancer is one of the most common cancers and is associated with high rates of morbidity and mortality. However, the introduction of immune checkpoint inhibitors (ICIs) has helped improve the prognosis of patients with lung cancer.^1^, ^2^, ^3^, ^4^, ^5^, ^6^ In addition to ICI monotherapy, combined treatment using an ICI and cytotoxic anticancer drugs has been approved as standard therapy for non‐small cell lung cancer based on results from clinical trials.^7^ Tumor cell expression of programmed death‐ligand 1 (PD‐L1) is a commonly used biomarker for predicting the response to ICI therapy. However, this biomarker has limited utility, as some patients with high PD‐L1 expression experience a poor response to ICI treatment, and it can be difficult in some cases to obtain a tumor specimen and evaluate PD‐L1 expression.^8^

The effects of ICI treatment are mediated by T cells, and T‐cell dysfunction can lead to treatment resistance.^9^ Interferon‐γ (IFN‐γ) is released from T cells as part of their normal activation, and IFN‐γ concentrations are reportedly related to ICI treatment response.^10^ Furthermore, disruption of the IFN‐γ receptor signaling pathway can lead to resistance to anti‐PD‐L1 therapy.^11^

An IFN‐γ releasing assay (IGRA) can be used to identify *Mycobacterium tuberculosis* based on T‐cell production of IFN‐γ in response to stimulation with tuberculosis‐specific antigens (TBAgs). As a positive control, the T‐cell response is quantified after nonspecific stimulation using phytohemagglutinin (PHA). Thus, the ability of T cells to produce IFN‐γ in response to PHA stimulation may reflect the immune status of individual patients.^12^ The T‐SPOT.TB test is a type of IGRA that evaluates T‐cell production of IFN‐γ using an enzyme‐linked immunospot count after antigen stimulation. Therefore, the present study evaluated whether the T‐SPOT test could be used to quantify IFN‐γ production by peripheral T cells and thus predict the response to ICI treatment in patients with non‐small cell lung cancer.

## MATERIALS AND METHODS

### Patients

This retrospective study evaluated data from adult patients with non‐small cell lung cancer who received ICI treatment at the Yokohama City University Medical Center, Minami Kyosai Hospital, and Yokohama City University Hospital between January 2016 and August 2019. The inclusion criteria were age >18 years, a diagnosis of stage IV or postoperative recurrence non‐small cell lung cancer, ICI treatment (nivolumab, pembrolizumab, or atezolizumab), and available IGRA test results, which had been used to screen for tuberculosis infection before starting anticancer chemotherapy in any line. The exclusion criteria were a diagnosis of small cell lung cancer and clear signs of infectious disease at the start of ICI treatment. Only the first ICI treatment was considered in patients who received multiple ICI treatments. Data regarding progression‐free survival (PFS), overall survival (OS), patient characteristics, pathological type, PD‐L1 tumor proportion score (TPS), the categorical T‐SPOT test result, and the number of immunospots in each T‐SPOT field were collected. The study's retrospective protocol was approved by our institutional review board (B191200043), which waived the requirement for informed consent.

### Treatment regimens

Patients were considered eligible if they had received ICIs as monotherapy or in combination with chemotherapy. Nivolumab monotherapy was administered intravenously at a dose of 3 mg/kg every 2 weeks. Pembrolizumab monotherapy was administered intravenously at a dose of 200 mg/kg every 3 weeks. Atezolizumab monotherapy was administered intravenously at a dose of 1200 mg/kg every 3 weeks. The combination regimens involved pembrolizumab with cisplatin/carboplatin and pemetrexed/paclitaxel.

### T‐SPOT.TB assay

The T‐SPOT tests were performed according to the manufacturer's protocol. Pretreatment peripheral blood samples were collected into heparinized tubes to isolate peripheral blood mononuclear cells (PBMCs). The isolated PBMCs were incubated in a microplate well on which IFN‐γ antibody was immobilized with or without TBAgs (ESAT‐6 or CFP‐10) or PHA for nonspecific stimulation as a positive control. About 2.5 × 10^5^ PBMCs were added to each well, and the incubation conditions were 37°C, and 5% CO_2_ for 16–20 h. After the incubation, the secondary antibody was added and allowed to react at 4°C for 1 h. The number of spots was counted in the T‐SPOT test, and the results were graded according to the categories described in the manual. The median number of T‐SPOT immunospots was used to categorize results as having high and low numbers of immunospots.

### Statistical analysis

Baseline patient characteristics were extracted from their medical records. The PFS interval was calculated from the start of ICI treatment to the first instance of disease progression (defined according to version 1.1 of the Response Evaluation Criteria in Solid Tumors) or death because of any cause. The OS interval was calculated from the start of ICI treatment to death or the last visit date. Curves for PFS and OS were compared using the Kaplan–Meier method and the log‐rank test. A Cox proportional hazards model was used for the multivariable analysis, and differences were considered statistically significant at *p* values <0.05. All analyses were performed using JMP Pro 15 software (SAS Institute Inc.).

## RESULTS

### Baseline characteristics

**FIGURE 1:**
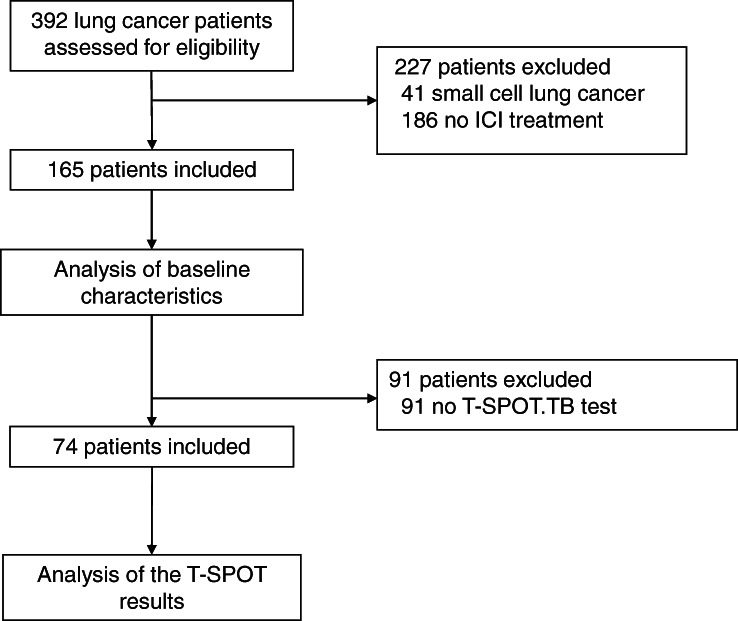
Study flowchart. A total of 392 lung cancer patients was assessed, although we excluded 41 patients with small cell lung cancer, 186 patients without immune checkpoint inhibitor (ICI) treatment, and 91 patients who did not undergo T‐SPOT.TB testing. As one patient received two ICI regimens, we only considered the first ICI treatment. Thus, 74 patients were included

A total of 392 lung cancer patients were assessed for eligibility (Figure 1). However, we excluded 41 patients with small cell lung cancer, 186 patients who did not receive ICI treatment, and 91 patients who did not undergo T‐SPOT testing. Thus, the study included data from 74 patients who received ICI treatment and had T‐SPOT test results.

The baseline characteristics are summarized in Table 1. The median age was 67 years (range 34–83 years) and 60 patients were men. Most patients had a good performance status (PS), with 38 patients having a PS of 0, 26 patients having a PS of 1, nine patients having a PS of 2, and one patient having a PS of 3. When ICI therapy was performed during the lung cancer treatment, 24 patients were treated in the first line, 26 patients in the second line, and 24 patients after third‐line treatments. The treatment regimens of ICI involved nivolumab monotherapy (34 patients), pembrolizumab monotherapy (30 patients), atezolizumab monotherapy (five patients), and pembrolizumab in combination with platinum‐based chemotherapy (five patients). The PD‐L1 TPS values were >50% for 23 patients, 1–49% for 15 patients, <1% for 14 patients, and unknown for 22 patients. The histological findings were adenocarcinoma in 47 patients (63.5%), squamous cell carcinoma in 20 patients (27.0%), and others in seven patients (9.5%). Ten patients (13.5%) had driver mutation‐positive lung cancer, which involved the epidermal growth factor receptor (EGFR) gene in seven patients, the echinoderm microtubule‐associated protein‐like 4 anaplastic lymphoma kinase fusion (EML4‐ALK) gene in two patients, and the Kirsten rat sarcoma viral oncogene homolog (KRAS) in one patient.

**TABLE 1:** Baseline characteristics

		Total (*N* = 74)	
		*N*	%
Age	<70 years	41	55.4
	≥70 years	33	44.6
Sex	Male	60	81.1
	Female	14	18.9
Performance status	0	38	51.3
	1	26	35.1
	2	9	12.2
	3	1	1.4
Treatment	Nivolumab	34	45.9
	Pembrolizumab	30	40.5
	Atezolizumab	5	6.8
	Combined with pembrolizumab and chemotherapy	5	6.8
Line	1	24	32.4
	2	26	35.1
	≥3	24	32.4
Pathology	Adenocarcinoma	47	63.5
	Squamous cell carcinoma	20	27.0
	Other	7	9.5
PD‐L1 TPS	<1%	14	18.9
	1–49%	15	20.3
	≥50%	23	31.1
	Unknown	22	29.7
Metastasis	Brain	15	20.3
	Liver	6	8.1
Driver gene mutation	Positive	10	13.5
	Negative	64	86.5

*Abbreviations*: PD‐L1, programmed death‐ligand 1; TPS, tumor proportion score.

### The T‐SPOT results

The categorical T‐SPOT results (judged according to the manufacturer's instructions) were negative for 63 patients (85.1%), positive for five patients (6.8%), intermediate for five patients (6.8%), and indeterminate for one patient (1.4%). No patients were diagnosed with active tuberculosis.

The numbers of spots per T‐SPOT field are shown in Table 2. Most patients had low levels of IFN‐γ release in response to ESAT‐6, CFP‐10, and the nil control. The median response to PHA (positive control) was 202 spots (range 38–737 spots).

**TABLE 2:** The T‐SPOT results from 74 cases

T‐SPOT results			
	*N*	%	
Negative	63	85.1	
Intermediate	5	6.8	
Positive	5	6.8	
Indeterminate	1	1.4	
**Numbers of spots in T‐SPOT field**			
	Median	Mean	SD
None	0	1.89	12.9
ESAT‐6	0	2.65	13.2
CFP‐10	0	3.22	12.4
PHA	202.5	263.4	19.3

*Abbreviations*: CFP‐10, a tuberculosis‐specific antigen; ESAT‐6, a tuberculosis‐specific antigen; PHA, phytohemagglutinin; SD, standard deviation.

### Correlation between spot counts and response to ICI treatment

**FIGURE 2:**
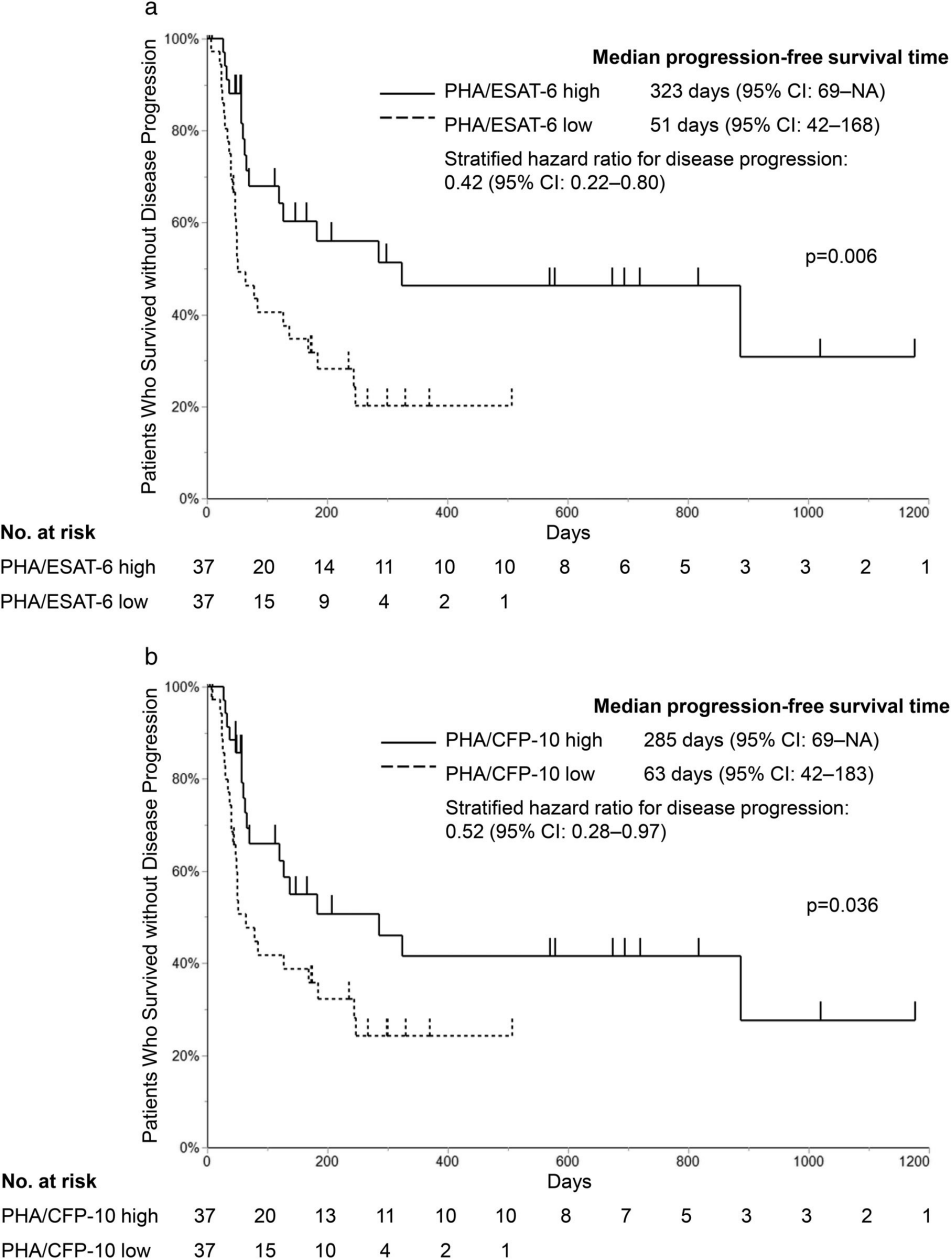
Kaplan–Meier analysis according to a low or high number of PHA/TBAg spots. Patients were divided according to the median numbers of spots for (a) PHA/ESAT‐6 and (b) PHA/CFP‐10. ESAT‐6, a tuberculosis‐specific antigen; CFP‐10, a tuberculosis‐specific antigen; PHA, phytohemagglutinin; NA, not available

We evaluated the patients' characteristics and spot counts for relationships with PFS. Patients were classified into two groups using the median number of spots in response to PHA divided by the median number of spots in response to the TBAgs. This division was performed to rectify an immune response to TBAgs. When there were no spots in response to TBAgs, we used the number of PHA spots as the denominator. Figure 2(a) shows the PFS curves when we categorized the PHA/ESAT‐6 spot counts as high or low (median PFS 323 days vs. 51 days; *p* = 0.006). Figure 2(b) shows the PFS curves when we categorized the PHA/CFP‐10 spot counts as high or low (median PFS 285 days vs. 63 days; *p* = 0.036). The patients in the PHA/TBAg high and low groups had generally similar characteristics, with the exception of unknown PD‐L1 status (Table 3). Given the larger number of patients with unknown PD‐L1 status in the PHA high group, multivariable Cox proportional hazards analysis was performed for PS, PD‐L1 TPS, and age (known predictors of response to ICI therapy and chemotherapy). The results revealed that better PFS was associated with better PS, higher PHA/ESAT‐6 spot count, and higher PD‐L1 TPS (Table 4). Similar to PFS, the analysis of the relationship between OS and PHA/TBAg spots, an increase in the number of PHA/TBAg spots were associated with a longer OS (Supporting Information Figure S1, and Supporting Information Tables S1 and S2).

**TABLE 3:** Patient characteristics according to the number of T‐SPOT.TB spots stimulated by PHA/TBAg

		PHA/ESAT‐6 spots			PHA/CFP‐10 spots		
		Low	High	*p*	Low	High	*p*
Age	<70 years	20	21	0.815	19	22	0.483
	≥70 years	17	16		18	15	
Sex	Male	29	31	0.552	30	30	1.000
	Female	8	6		7	7	
Performance status	0	20	18	0.372	21	17	0.149
	1	11	15		9	17	
	2	6	3		6	3	
	3	0	1		1	0	
Treatment	Nivolumab	15	19	0.703	16	18	0.884
	Pembrolizumab	17	13		16	14	
	Atezolizumab	3	2		2	3	
	Combined with pembrolizumab and chemotherapy	2	3		3	2	
Line	1	13	11	0.609	13	11	0.317
	2	14	12		15	11	
	≥3	10	14		9	15	
Histology	Adenocarcinoma	23	24	0.091	23	24	0.091
	Squamous cell carcinoma	8	12		8	12	
	Other	6	1		6	1	
PD‐L1 TPS	<1%	11	3	0.070	10	4	0.122
	1–49%	10	5		10	5	
	>50%	11	12		11	12	
	Unknown	5	17	0.002	6	16	0.001
Metastasis	Brain	7	8	0.772	8	7	0.772
	Liver	4	2	0.390	4	2	0.390
Driver mutation	Positive	4	6	0.495	3	7	0.169

*Abbreviations*: CFP‐10, a tuberculosis‐specific antigen; ESAT‐6, a tuberculosis‐specific antigen; PD‐L1, programmed death‐ligand 1; PHA, phytohemagglutinin; TBAg, tuberculosis‐specific‐antigen; TPS, tumor proportion score.

**TABLE 4:** Multivariable Cox proportional hazards analysis of progression‐free survival

	Category	HR	95% CI	*p* (log‐rank)
PHA/ESAT‐6	≥178 (vs. <178)	0.49	0.25–0.96	0.036
PS	0–1 (vs. 2–3)	0.20	0.07–0.53	0.012
PD‐L1 TPS	≥50% (vs. <50% or NA)	0.44	0.21–0.92	0.029
Age	<70 years (vs. ≥70 years)	1.30	0.69–2.42	0.406

*Abbreviations*: CI, confidence interval; ESAT‐6, a tuberculosis‐specific antigen; HR, hazard ratio; NA, not available; PD‐L1, programmed death‐ligand 1; PHA, phytohemagglutinin; PS, performance status; TPS, tumor proportion score.

### The PHA‐induced spot count independently predicts PFS among patients with negative T‐SPOT results

**FIGURE 3:**
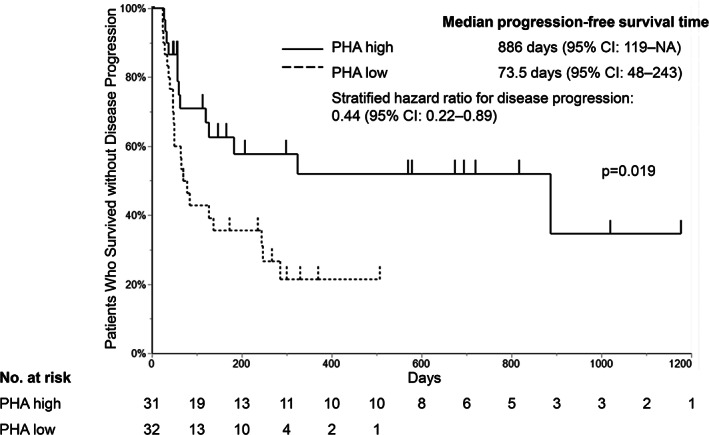
Kaplan–Meier analysis according to a low or high number of PHA spots among patients with negative T‐SPOT results. Patients were divided according to the median number of phytohemagglutinin (PHA) spots. NA, not available

Given that T‐cell response to TBAgs might be involved in their response to mitogen activation, we excluded patients with positive, intermediate, and indeterminate T‐SPOT results and only considered patients with negative T‐SPOT results. Patients in the PHA high and low groups had similar characteristics, except for PD‐L1 status (Table 5). The PFS outcomes were compared between the PHA high and low groups (Figure 3), which revealed that the PHA high group had significantly longer median PFS (886 days vs. 73.5 days; *p* = 0.019). Multivariate analysis was performed because the PD‐L1 status differed between the two groups (Table 6), and the results showed that better PFS was associated with better PS and a high PHA spot count. Similarly for OS, a high number of PHA spots was associated with better OS (Supporting Information Figure S2 and Table S3).

**TABLE 5:** Patient characteristics according to the number of spots in T‐SPOT‐negative patients

		PHA spots		*p*
		Low	High	
Age	<70 years	18	19	0.685
	≥70 years	14	12	
Sex	Male	25	25	0.805
	Female	7	6	
Performance status	0	19	15	0.179
	1	8	14	
	2	5	2	
Treatment	Nivolumab	14	15	0.901
	Pembrolizumab	14	11	
	Atezolizumab	2	2	
	Combined with pembrolizumab and chemotherapy	2	3	
Line	1	13	9	0.347
	2	11	9	
	≥3	8	13	
Histology	Adenocarcinoma	17	24	0.124
	Squamous cell carcinoma	10	7	
	Other	5	0	
PD‐L1 TPS	<1%	9	3	0.329
	1–49%	7	5	
	>50%	11	10	
	Unknown	5	13	0.020
Metastasis	Brain	3	7	0.147
	Liver	4	1	0.159
Driver mutation	Positive	3	6	0.254

*Abbreviations*: PD‐L1, programmed death‐ligand 1; PHA, phytohemagglutinin; TPS, tumor proportion score.

**TABLE 6:** Multivariable Cox proportional hazards analysis of progression‐free survival in T‐SPOT‐negative patients

Parameter	Category	HR	95% CI	*p* (log‐rank)
PHA	≥196 (vs. <196)	0.46	0.22–0.96	0.039
PS	0–1 (vs. 2–3)	0.10	0.03–0.35	<0.001
PD‐L1 TPS	≥50% (vs. <50% or NA)	0.53	0.25–1.16	0.114
Age	<70 years (vs. ≥70 years)	1.26	0.63–2.53	0.509

*Abbreviations*: CI, confidence interval; HR hazard ratio; NA, not available; PD‐L1, programmed death‐ligand 1; PHA, phytohemagglutinin; PS, performance status; TPS, tumor proportion score.

## DISCUSSION

The increasing use of ICI treatment for lung cancer patients highlights the need for biomarkers that accurately predict treatment response, which can help minimize ineffective treatment and prevent adverse effects that are often severe. This study evaluated whether the number of IFN‐γ‐releasing cells based on T‐SPOT counts could predict the response to ICI treatment, and we observed that the PHA/TBAg spot count (number of IFN‐γ‐releasing cells in response to PHA/TBAg stimulation) was significantly associated with PFS after starting ICI treatment for non‐small cell lung cancer (Figure 2 and Table 4). Moreover, among patients with negative T‐SPOT results, a high PHA spot count was associated with significantly better PFS (vs. a low PHA spot count) (Figure 3 and Table 6). These results showed a similar trend for OS (Supporting Information Figure S1, S2 and Table S1‐S3).

Interferon‐γ is an important factor that can exert broad pro‐ or antitumor effects on the immune response.^13^ For example, IFN‐γ upregulates the MHC class I beta‐2‐microglobulin in tumor cells^14^, ^15^ and IFN‐γ also improves the antitumor immune response of NK cells and cytotoxic T‐lymphocytes.^16^, ^17^, ^18^ Nevertheless, another group has reported that IFN‐γ can downregulate the antitumor immune response by promoting regulatory T‐cell (Treg) function and suppressing effector T‐cell function via enhancement of indoleamine 2,3‐dioxygenase secretion from cancer cells and antigen‐presenting cells.^19^ Cho et al. have also reported that IFN‐γ activates the nonclassical MHC class Ia genes and causes evasion of cytotoxic lymphocytes in melanoma.^20^ In the context of the PD‐1/PD‐L1 axis, IFN‐γ induces PD‐L1 expression on both cancer cells and local immune cells.^21^, ^22^ Thus, IFN‐γ appears to play two opposing but important roles in regulating the antitumor immune response.

Our results indicate that the number of IFN‐γ‐releasing peripheral T cells, based on T‐SPOT counts, might be useful for predicting the response to ICI treatment among patients with non‐small cell lung cancer. In this context, patients who have T cells that produce large amounts of IFN‐γ might be more likely to experience a favorable response to ICI treatment. Huang et al. evaluated 84 patients who received cytotoxic therapy for lung cancer and reported that higher IFN‐γ production after PHA stimulation was associated with better outcomes,^23^ although we are only aware of two small studies that have evaluated the relationship between IFN‐γ production and response to ICI treatment. Karachaliou et al. evaluated serum mRNA expression of IFN‐γ before nivolumab treatment, which revealed that high pretreatment expression was associated with significantly longer median PFS (high group [*n* = 11] 5.1 months vs. low group [*n* = 4] 2.0 months; *p* = 0.0124).^24^ Kanai et al. used the QuantiFERON‐TB Gold Plus (QFT‐Plus, Qiagen) assay, which evaluates IFN‐γ levels using ELISA,^10^ and reported that a significantly longer PFS was observed for the high IFN‐γ group (*n* = 24) relative to the low IFN‐γ group (*n* = 5). Our data, which were derived from a larger sample of patients, strongly support the relationship between high IFN‐γ production and prolonged PFS in this setting. Our study was based on the T‐SPOT test, which evaluates IFN‐γ production based on immunospots after PBMCs are exposed to different antigens. The T‐SPOT results are adjusted for the peripheral blood lymphocyte count, which can help reduce the influence of varying lymphocyte counts between patients.^25^

There is evidence that ICI treatment is associated with a risk of developing tuberculosis,^26^, ^27^, ^28^ and we observed positive T‐SPOT results in 6.8% of our patients who received ICI treatment for non‐small cell lung cancer (Table 2). However, this proportion is comparable to the proportion of positive results that we previously reported among all outpatients at our institution (8.4%).^29^ Although none of our patients developed active tuberculosis during or after ICI treatment, ICI is still considered a risk factor for the development and aggravation of tuberculosis.^30^ Therefore, it may be prudent to consider prophylactic treatment for patients with positive T‐SPOT results.

In this study, the timing of the T‐spot test was not limited to before the first line. A report on T‐SPOT tests^29^ has confirmed the reproducibility of its results and the timing of blood collection might not be a problem. To examine the details, a unified study of the timing of blood collection should be considered.

This study has several limitations that should be considered. First, we only considered a small sample of patients from a few Japanese institutions. Second, the retrospective study design is prone to bias and incomplete data. Third, the T‐SPOT results reflect the reaction of PBMCs, and further studies are needed to confirm the relationship between the tumor microenvironment and peripheral blood findings.

In conclusion, the present study revealed that based on the T‐SPOT test results, the number of IFN‐γ‐releasing peripheral T cells after PHA stimulation might be useful for predicting the response to ICI treatment among patients with non‐small cell lung cancer. Thus, a larger prospective study is warranted to confirm whether the response of peripheral blood‐derived T cells to PHA is a useful biomarker for predicting the response to ICI treatment.

## CONFLICTS OF INTEREST

The authors have no conflicts of interest to declare.

## Supporting information


Supporting Information Figure S1

Supporting Information Table S1

## References

[1] Cancer Information Service , National Cancer Center, Japan (Vital Statistics of Japan). Cancer Registry and Statistics.

[2] Borghaei H , Paz‐Ares L , Horn L , Spigel DR , Steins M , Ready NE , et al. Nivolumab versus docetaxel in advanced nonsquamous non‐small‐cell lung cancer. N Engl J Med. 2015;373:1627–39. 26412456 10.1056/NEJMoa1507643 PMC5705936

[3] Brahmer J , Reckamp KL , Baas P , Crinò L , Eberhardt WEE , Poddubskaya E , et al. Nivolumab versus docetaxel in advanced squamous‐cell non‐small‐cell lung cancer. N Engl J Med. 2015;373:123–35. 26028407 10.1056/NEJMoa1504627 PMC4681400

[4] Reck M , Rodríguez‐Abreu D , Robinson AG , Hui R , Csőszi T , Fülöp A , et al. Pembrolizumab versus chemotherapy for PD‐L1‐positive non‐small‐cell lung cancer. N Engl J Med. 2016;375:1823–33. 27718847 10.1056/NEJMoa1606774

[5] Rittmeyer A , Barlesi F , Waterkamp D , Park K , Ciardiello F , von Pawel J , et al. Atezolizumab versus docetaxel in patients with previously treated non‐small‐cell lung cancer (OAK): a phase 3, open‐label, multicentre randomised controlled trial. Lancet. 2017;389:255–65. 27979383 10.1016/S0140-6736(16)32517-X PMC6886121

[6] Zhou GW , Xiong Y , Chen S , Xia F , Li Q , Hu J . Anti‐PD‐1/PD‐L1 antibody therapy for pretreated advanced nonsmall‐cell lung cancer: a meta‐analysis of randomized clinical trials. Medicine. 2016;95:e4611. 27583876 10.1097/MD.0000000000004611 PMC5008560

[7] Gandhi L , Rodríguez‐Abreu D , Gadgeel S , Esteban E , Felip E , De Angelis F , et al. Pembrolizumab plus chemotherapy in metastatic non‐small‐cell lung cancer. N Engl J Med. 2018;378:2078–92. 29658856 10.1056/NEJMoa1801005

[8] Ilié M , Hofman P . Pros: can tissue biopsy be replaced by liquid biopsy? Transl Lung Cancer Res. 2016;5:420–3. 27655109 10.21037/tlcr.2016.08.06 PMC5009092

[9] Jenkins RW , Barbie DA , Flaherty KT . Mechanisms of resistance to immune checkpoint inhibitors. Br J Cancer. 2018;118:9–16. 29319049 10.1038/bjc.2017.434 PMC5765236

[10] Kanai T , Suzuki H , Yoshida H , Matsushita A , Kawasumi H , Samejima Y , et al. Significance of quantitative interferon‐gamma levels in non‐small‐cell lung cancer patients' response to immune checkpoint inhibitors. Anticancer Res. 2020;40:2787–93. 32366425 10.21873/anticanres.14251

[11] Shin DS , Zaretsky JM , Escuin‐Ordinas H , Garcia‐Diaz A , Hu‐Lieskovan S , Kalbasi A , et al. Primary resistance to PD‐1 blockade mediated by JAK1/2 mutations. Cancer Discov. 2017;7:188–201. 27903500 10.1158/2159-8290.CD-16-1223 PMC5296316

[12] Belliere J , Blancher A . QuantiFERON test interpretation in patients receiving immunosuppressive agents: an alert. Eur Respir J. 2017;49:1602102. 28381433 10.1183/13993003.02102-2016

[13] Zaidi MR . The interferon‐gamma paradox in cancer. J Interferon Cytokine Res. 2019;39:30–8. 30388040 10.1089/jir.2018.0087 PMC6350411

[14] Martini M , Testi MG , Pasetto M , Cristina Picchio M , Innamorati G , Mazzocco M , et al. IFN‐γ‐mediated upmodulation of MHC class I expression activates tumor‐specific immune response in a mouse model of prostate cancer. Vaccine. 2010;28:3548–57. 20304037 10.1016/j.vaccine.2010.03.007

[15] Teranishi S , Kobayashi N , Katakura S , Kamimaki C , Kubo S , Shibata Y , et al. Class A CpG oligodeoxynucleotide inhibits IFN‐γ‐induced signaling and apoptosis in lung cancer. Thorac Cancer. 2020;11:983–92. 32067413 10.1111/1759-7714.13351 PMC7113052

[16] Senik A , Stefanos S , Kolb JP , Lucero M , Falcoff E . Enhancement of mouse natural killer cell activity by type II interferon. Ann Immunol (Paris). 1980;131C:349–61. 6157349

[17] Ivashkiv LB . IFNγ: signalling, epigenetics and roles in immunity, metabolism, disease and cancer immunotherapy. Nat Rev Immunol. 2018;18:545–58. 29921905 10.1038/s41577-018-0029-z PMC6340644

[18] Kang S , Brown HM , Hwang S . Direct antiviral mechanisms of interferon‐gamma. Immune Netw. 2018;18:e33. 30402328 10.4110/in.2018.18.e33 PMC6215902

[19] Katz JB , Muller AJ , Prendergast GC . Indoleamine 2,3‐dioxygenase in T‐cell tolerance and tumoral immune escape. Immunol Rev. 2008;222:206–21. 18364004 10.1111/j.1600-065X.2008.00610.x

[20] Cho HI , Celis E . Optimized peptide vaccines eliciting extensive CD8 T‐cell responses with therapeutic anti‐tumor effects. Cancer Res. 2009;69:9012–9. 19903852 10.1158/0008-5472.CAN-09-2019 PMC2789207

[21] Garcia‐Diaz A , Shin DS , Moreno BH , Saco J , Escuin‐Ordinas H , Abril Rodriguez G , et al. Interferon receptor signaling pathways regulating PD‐L1 and PD‐L2 expression. Cell Rep. 2017;19:1189–11201. 28494868 10.1016/j.celrep.2017.04.031 PMC6420824

[22] Peng Q , Qiu X , Zhang Z , Zhang S , Zhang Y , Liang Y , et al. PD‐L1 on dendritic cells attenuates T cell activation and regulates response to immune checkpoint blockade. Nat Commun. 2020;11:4835. 32973173 10.1038/s41467-020-18570-x PMC7518441

[23] Huang HC , Su WJ , Chiang CL , Feng J‐Y , Huang H‐Y , Lin C‐H , et al. The predictive value of the interferon‐γ release assay for chemotherapy responses in patients with advanced non‐small‐cell lung cancer. Lung Cancer. 2018;115:64–70. 29290264 10.1016/j.lungcan.2017.11.016

[24] Karachaliou N , Gonzalez‐Cao M , Crespo G , Drozdowskyj A , Aldeguer E , Gimenez‐Capitan A , et al. Interferon gamma, an important marker of response to immune checkpoint blockade in non‐small cell lung cancer and melanoma patients. Ther Adv Med Oncol. 2018;10:1758834017749748. 29383037 10.1177/1758834017749748 PMC5784541

[25] Komiya K , Ariga H , Nagai H , Teramoto S , Kurashima A , Shoji S , et al. Impact of peripheral lymphocyte count on the sensitivity of 2 IFN‐gamma release assays, QFT‐G and ELISPOT, in patients with pulmonary tuberculosis. Intern Med. 2010;49:1849–55. 20823644 10.2169/internalmedicine.49.3659

[26] van Eeden R , Rapoport BL , Smit T , Anderson R . Tuberculosis infection in a patient treated with nivolumab for non‐small cell lung cancer: case report and literature review. Front Oncol. 2019;9:659. 31396484 10.3389/fonc.2019.00659 PMC6668214

[27] Tezera LB , Bielecka MK , Ogongo P , Walker NF , Ellis M , Garay‐Baquero DJ , et al. Anti‐PD‐1 immunotherapy leads to tuberculosis reactivation via dysregulation of TNF‐α. Elife. 2020;9:e52668. 32091388 10.7554/eLife.52668 PMC7058383

[28] Fujita K , Terashima T , Mio T . Anti‐PD1 antibody treatment and the development of acute pulmonary tuberculosis. J Thorac Oncol. 2016;11:2238–40. 27423391 10.1016/j.jtho.2016.07.006

[29] Teranishi S , Kobayashi N , Aoki A , Katakura S , Yamamoto M , Koizumi H , et al. Reproducibility of the T‐SPOT.TB test for screening *Mycobacterium tuberculosis* infection in Japan. J Infect Chemother. 2020;26:194–8. 31495568 10.1016/j.jiac.2019.08.006

[30] Anastasopoulou A , Ziogas DC , Samarkos M , Kirkwood JM , Gogas H . Reactivation of tuberculosis in cancer patients following administration of immune checkpoint inhibitors: current evidence and clinical practice recommendations. J Immunother Cancer. 2019;7:239. 31484550 10.1186/s40425-019-0717-7 PMC6727332

