# Supplementary material for: T‐cell response to phytohemagglutinin in the interferon‐γ release assay as a potential biomarker for the response to immune checkpoint inhibitors in patients with non‐small cell lung cancer
Source: Thorac Cancer. 2021 May 4;12(11):1726–34. doi: 10.1111/1759-7714.13978 (PMC8169292; doi:10.1111/1759-7714.13978)
Supplement: Supplementary file 1 — Supporting Information Figure S1 [file TCA-12-1726-s002.pptx]

## Slide 1
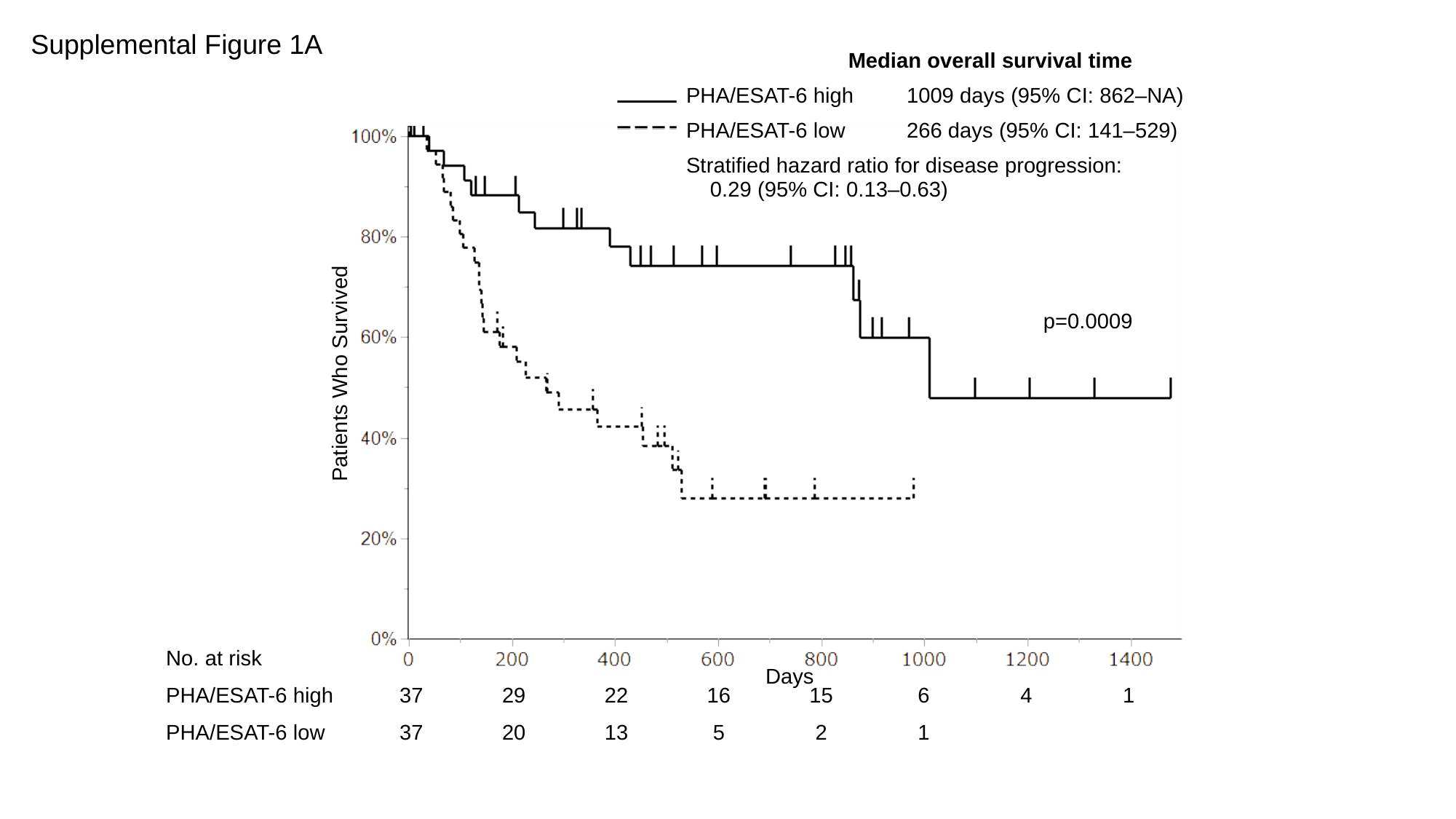

Supplemental Figure 1A
| Median overall survival time | |
| --- | --- |
| PHA/ESAT-6 high | 1009 days (95% CI: 862–NA) |
| PHA/ESAT-6 low | 266 days (95% CI: 141–529) |
| Stratified hazard ratio for disease progression: 0.29 (95% CI: 0.13–0.63) | |
p=0.0009
Patients Who Survived
| No. at risk | | | | | | | | |
| --- | --- | --- | --- | --- | --- | --- | --- | --- |
| PHA/ESAT-6 high | 37 | 29 | 22 | 16 | 15 | 6 | 4 | 1 |
| PHA/ESAT-6 low | 37 | 20 | 13 | 5 | 2 | 1 | | |
Days

## Slide 2
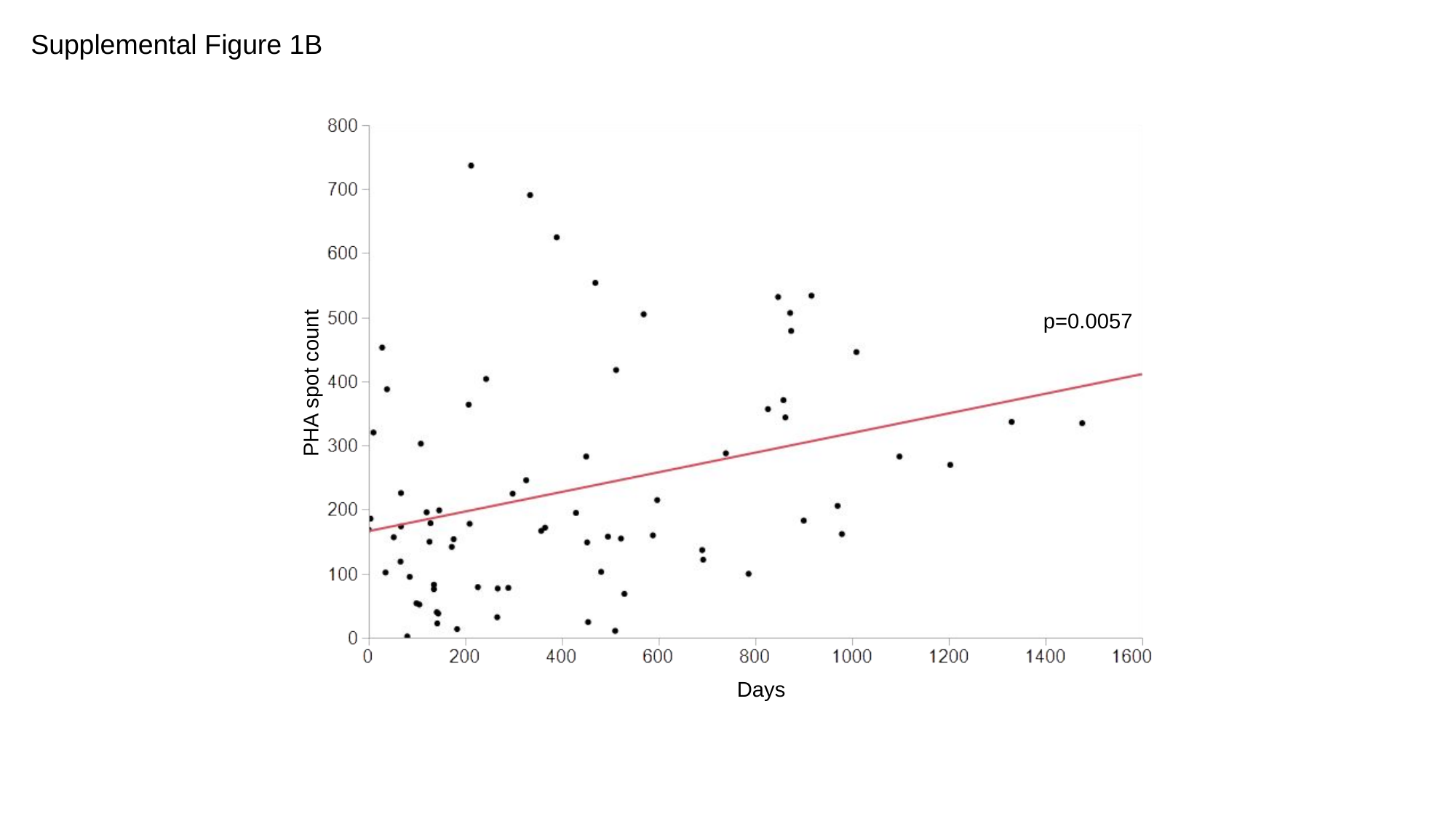

Supplemental Figure 1B
p=0.0057
PHA spot count
Days

## Slide 3
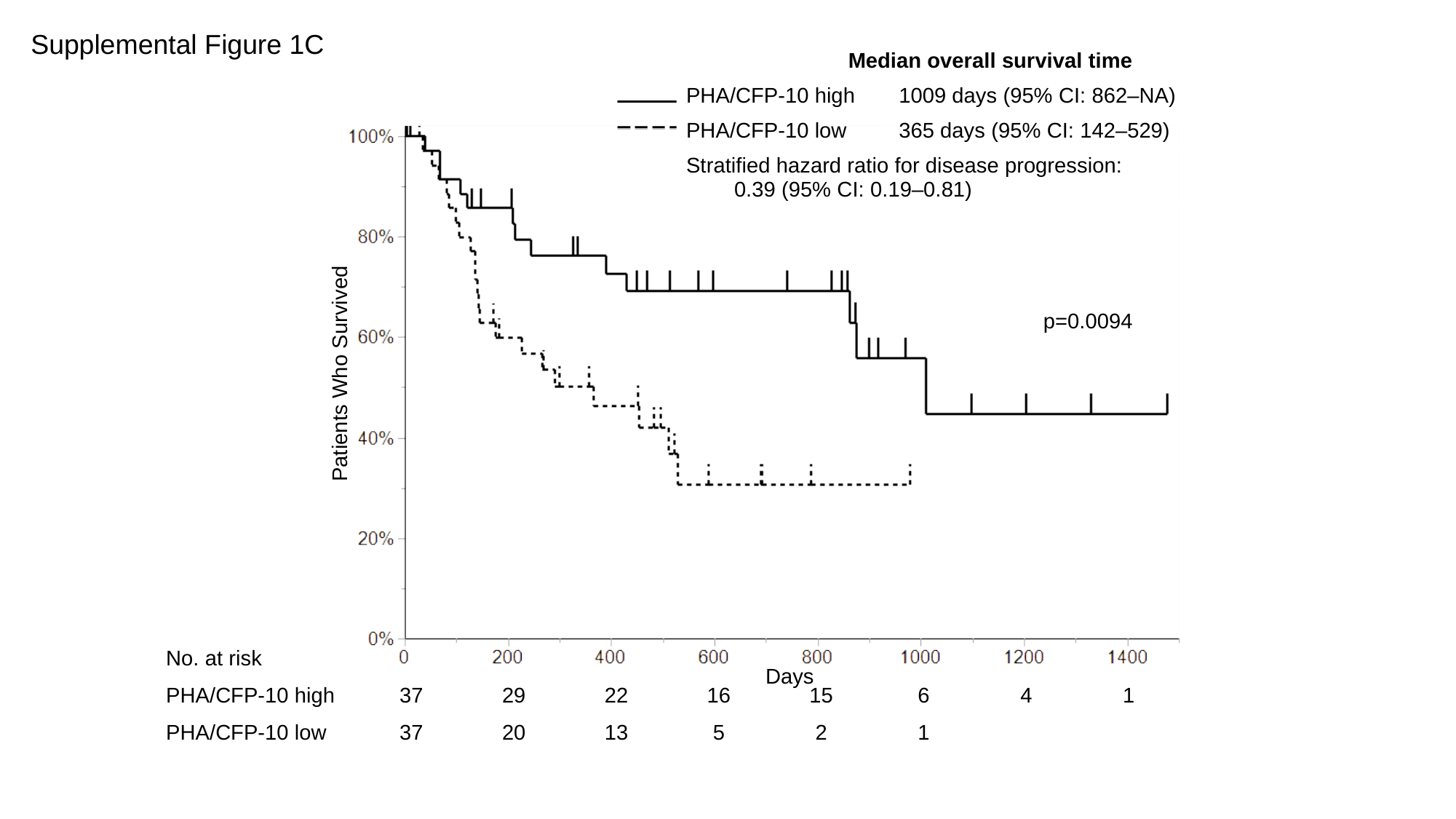

Supplemental Figure 1C
| Median overall survival time | |
| --- | --- |
| PHA/CFP-10 high | 1009 days (95% CI: 862–NA) |
| PHA/CFP-10 low | 365 days (95% CI: 142–529) |
| Stratified hazard ratio for disease progression: 0.39 (95% CI: 0.19–0.81) | |
p=0.0094
Patients Who Survived
| No. at risk | | | | | | | | |
| --- | --- | --- | --- | --- | --- | --- | --- | --- |
| PHA/CFP-10 high | 37 | 29 | 22 | 16 | 15 | 6 | 4 | 1 |
| PHA/CFP-10 low | 37 | 20 | 13 | 5 | 2 | 1 | | |
Days

## Slide 4
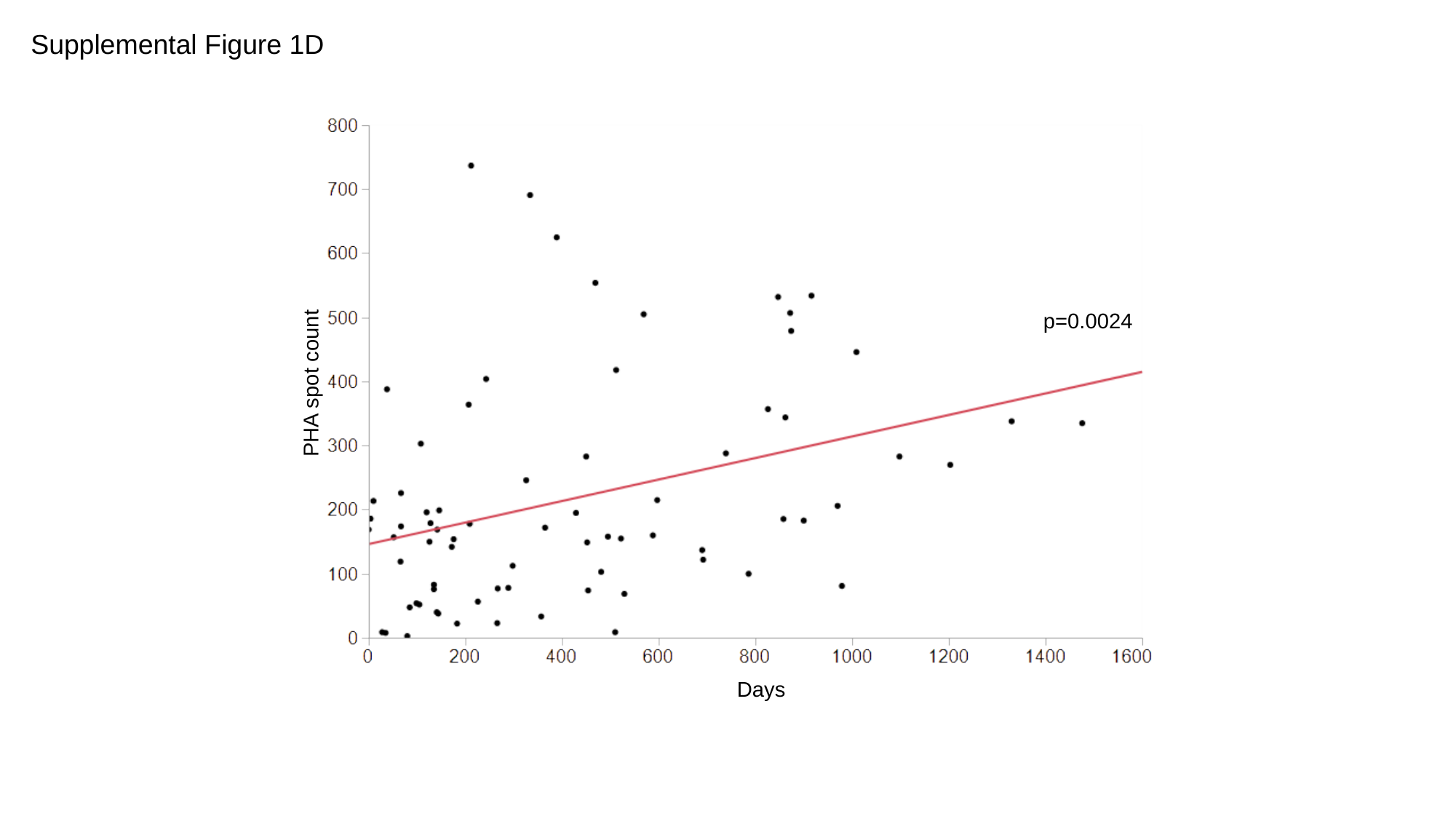

Supplemental Figure 1D
p=0.0024
PHA spot count
Days

## Slide 5
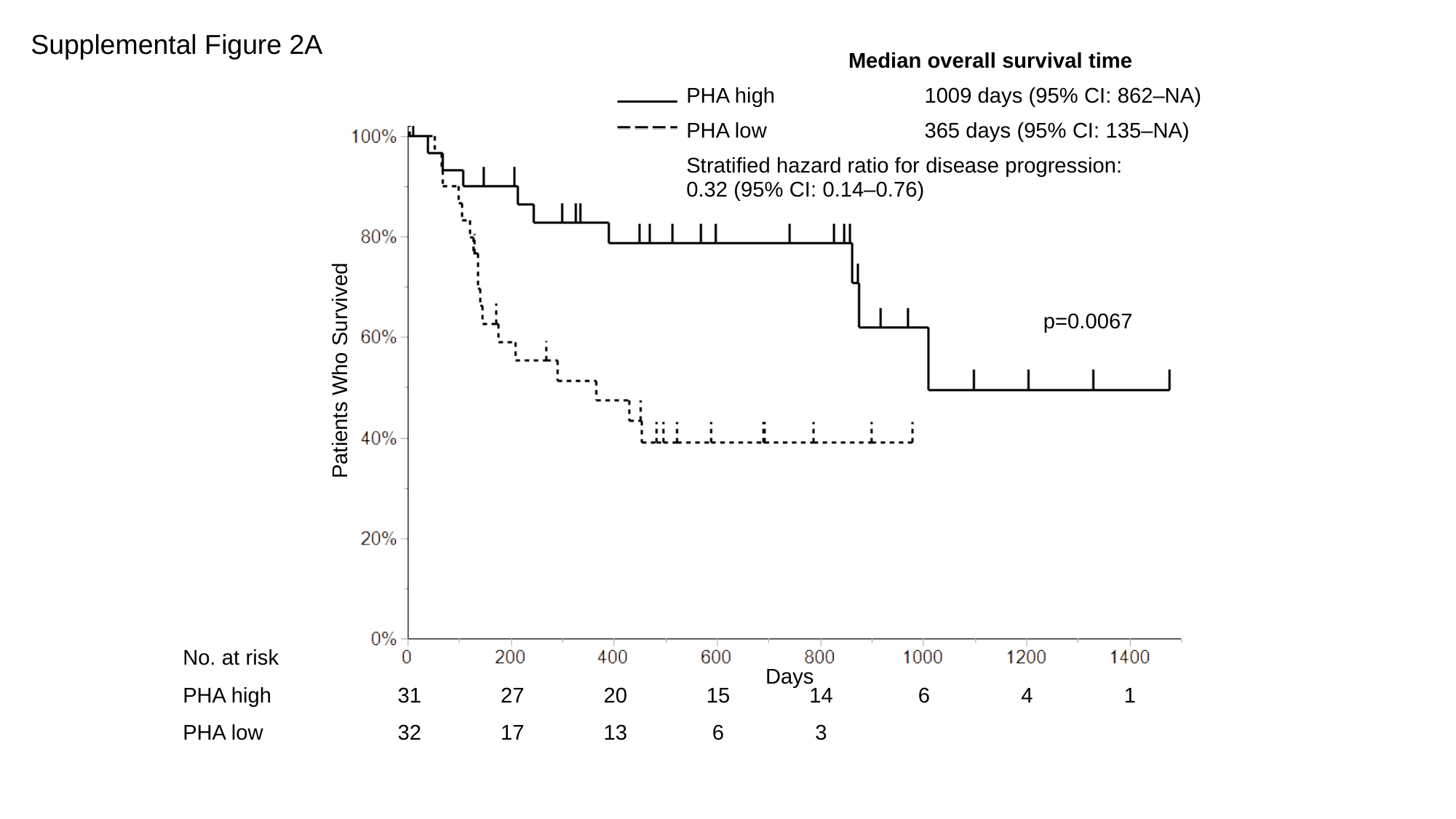

Supplemental Figure 2A
| Median overall survival time | |
| --- | --- |
| PHA high | 1009 days (95% CI: 862–NA) |
| PHA low | 365 days (95% CI: 135–NA) |
| Stratified hazard ratio for disease progression: 0.32 (95% CI: 0.14–0.76) | |
p=0.0067
Patients Who Survived
| No. at risk | | | | | | | | |
| --- | --- | --- | --- | --- | --- | --- | --- | --- |
| PHA high | 31 | 27 | 20 | 15 | 14 | 6 | 4 | 1 |
| PHA low | 32 | 17 | 13 | 6 | 3 | | | |
Days

## Slide 6
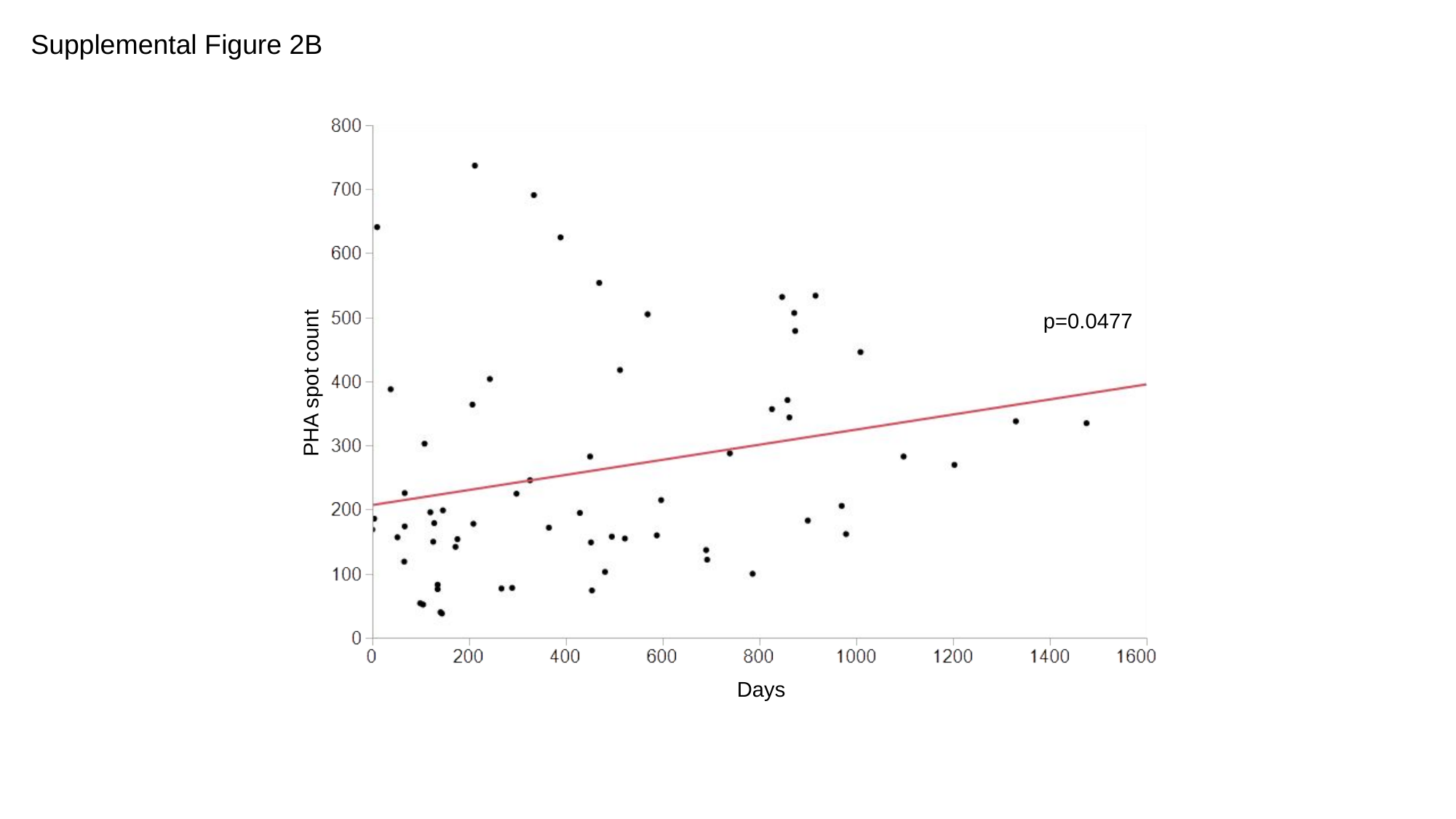

Supplemental Figure 2B
p=0.0477
PHA spot count
Days
