# Supplementary material for: T‐cell response to phytohemagglutinin in the interferon‐γ release assay as a potential biomarker for the response to immune checkpoint inhibitors in patients with non‐small cell lung cancer
Source: Thorac Cancer. 2021 May 4;12(11):1726–34. doi: 10.1111/1759-7714.13978 (PMC8169292; doi:10.1111/1759-7714.13978)
Supplement: Supplementary file 2 — Supporting Information Table S1 [file TCA-12-1726-s001.docx]

**Supplemental Table 1** Multivariable Cox proportional hazards analysis of overall survival

|  | **Category** | **HR** | **95% CI** | ***P* (log-rank)** |
| --- | --- | --- | --- | --- |
| PHA/ESAT-6 | ≥178 (vs. <178) | 0.31 | 0.14–0.68 | 0.003 |
| PS | 0–1 (vs. 2–3) | 0.13 | 0.04–0.39 | <0.001 |
| PD-L1 TPS | ≥50% (vs. <50% or NA) | 0.60 | 0.26–1.34 | 0.216 |
| Age | <70 years (vs. ≥70 years) | 3.07 | 1.36–6.93 | 0.007 |

PS, performance status; PHA, phytohemagglutinin; ESAT-6, a tuberculosis-specific antigen; PD-L1, programmed death-ligand 1; TPS, tumor proportion score; NA, not available.

**Supplemental Table 2** Multivariable Cox proportional hazards analysis of overall survival

|  | **Category** | **HR** | **95% CI** | ***P* (log-rank)** |
| --- | --- | --- | --- | --- |
| PHA/CFP-10 | ≥173 (vs. <173) | 0.41 | 0.19–0.87 | 0.022 |
| PS | 0–1 (vs. 2–3) | 0.13 | 0.04–0.38 | <0.001 |
| PD-L1 TPS | ≥50% (vs. <50% or NA) | 0.60 | 0.27–1.35 | 0.219 |
| Age | <70 years (vs. ≥70 years) | 3.02 | 1.35–6.76 | 0.007 |

PS, performance status; PHA, phytohemagglutinin; CFP-10, a tuberculosis-specific antigen; PD-L1, programmed death-ligand 1; TPS, tumor proportion score; NA, not available.

**Supplemental Table 3** Multivariable Cox proportional hazards analysis of overall survival in T-SPOT-negative patients

| **Parameter** | **Category** | **HR** | **95% CI** | ***P* (log-rank)** |
| --- | --- | --- | --- | --- |
| PHA | ≥196 (vs. <196) | 0.26 | 0.11–0.65 | 0.004 |
| PS | 0–1 (vs. 2–3) | 0.07 | 0.02–0.26 | <0.001 |
| PD-L1 TPS | ≥50% (vs. <50% or NA) | 0.73 | 0.30–1.78 | 0.490 |
| Age | <70 years (vs. ≥70 years) | 4.44 | 1.66–11.8 | 0.003 |

PS, performance status; PHA, phytohemagglutinin; PD-L1, programmed death-ligand 1; TPS, tumor proportion score; NA, not available.
